# Differential Evolutionary Constraints in the Evolution of Chemoreceptors: A Murine and Human Case Study

**DOI:** 10.1155/2014/696485

**Published:** 2014-01-23

**Authors:** Ricardo D'Oliveira Albanus, Rodrigo Juliani Siqueira Dalmolin, José Luiz Rybarczyk-Filho, Mauro Antônio Alves Castro, José Cláudio Fonseca Moreira

**Affiliations:** ^1^Departamento de Bioquímica, Universidade Federal do Rio Grande do Sul, Rua Ramiro Barcelos 2600, 90040-180 Porto Alegre, RS, Brazil; ^2^Departamento de Física e Biofísica, Universidade Estadual Paulista, Distrito de Rubião Júnior, S/N, 18618-970 Botucatu, SP, Brazil

## Abstract

Chemoreception is among the most important sensory modalities in animals. Organisms use the ability to perceive chemical compounds in all major ecological activities. Recent studies have allowed the characterization of chemoreceptor gene families. These genes present strikingly high variability in copy numbers and pseudogenization degrees among different species, but the mechanisms underlying their evolution are not fully understood. We have analyzed the functional networks of these genes, their orthologs distribution, and performed phylogenetic analyses in order to investigate their evolutionary dynamics. We have modeled the chemosensory networks and compared the evolutionary constraints of their genes in *Mus musculus*, *Homo sapiens*, and *Rattus norvegicus*. We have observed significant differences regarding the constraints on the orthologous groups and network topologies of chemoreceptors and signal transduction machinery. Our findings suggest that chemosensory receptor genes are less constrained than their signal transducing machinery, resulting in greater receptor diversity and conservation of information processing pathways. More importantly, we have observed significant differences among the receptors themselves, suggesting that olfactory and bitter taste receptors are more conserved than vomeronasal receptors.

## 1. Introduction

The ability to evaluate the environment has always been of vital importance to all organisms. In order to find food, detect dangers, and search for reproductive partners, a constant appraisal of the outside world must be made by any organism. Chemosensory reception is one such tool for this task, and it is present in all life forms. Over the last decade, several studies were conducted in order to characterize the different chemosensory receptors (CR) genes [1–4]. In vertebrates, they are coded by six major multigene families: the trace amine-associated receptors (TAAR) [5], the olfactory receptors (OR) [6], the type I and II vomeronasal receptors (V1R and V2R) [3, 4, 7], and type I and II taste receptors (T1R and T2R) [1, 2]. All proteins coded by these genes are G protein-coupled proteins [8].

Different from other environmental appraisal systems such as vision and hearing, which remained relatively stable once they were formed, chemosensory reception must be constantly tuned to an ever-changing environment of odors and toxins. This need for variability is reflected in the organization of the CR genes in the genome. In all studied species, it was found that these genes occur in great numbers, and there are considerable numbers of CR pseudogenes [4, 9–11], suggesting that they are prone to duplication and inactivation events. There are theories to explain the evolution of CR genes [9, 11–14], but several gaps regarding this subject still remain. For instance, there are no currently available data regarding the evolutionary dynamics of the chemosensory apparatus as a whole (i.e., the CR and its signal transducing machinery). Equally unclear are the differences in evolutionary dynamics among the CR families.

In this work, we have tackled the evolution of the mammalian CR gene families and their signal transducing machinery from a systems biology-oriented approach. We have analyzed the orthologs distribution of the chemosensory machinery, their functional networks topologies, and their phylogenetic diversity in *Mus musculus*, *Rattus norvegicus*, and *Homo sapiens*. We have found evidences that there are distinct evolutionary dynamics in the CR genes and the signal transducing apparatus. More importantly, we have observed significant differences among the CR gene families, suggesting distinct evolutionary dynamics for each receptor type.

## 2. Methods

### 2.1. Data Collection

In order to determine which receptors are involved in each sensory modality, we have gathered data from the Gene Ontology (GO) Consortium [15] regarding *Homo sapiens, Mus musculus, *and* Rattus norvegicus*. GO groups used were 0004984—MF Olfactory receptor activity, 0007608—BP Sensory perception of smell, 0008527—MF Taste receptor activity, 0050909—BP Sensory perception of taste, 0016503—MF Pheromone receptor activity, 0019236—BP Response to Pheromone. We have chosen these three species for our study due to robustness of their genomic/proteomic data available in databases. Studied genes were sorted in groups according to their receptor modality: olfactory receptors; taste receptors; and vomeronasal receptors. We made one further division of the GO taste group to separate taste receptors type 1 and 2 and study them separately because of their functional differences. TAAR genes were withdrawn from our analysis due to lack of data in the databases. Also due to lack of available data, we have combined the two vomeronasal families (V1R and V2R) and studied them as a single group (VN). We have sorted all genes in GO groups into two functional categories: the first consisted of genes coding the proteins directly involved in binding chemical stimuli (the chemosensory receptors *per se*), and the second consisted of the rest of the genes related to signal transduction machinery (STM).

Functional network parameters of proteins coded by CR genes were gathered using STRING database (String-DB), version 8.3 [16], using their corresponding ENSEMBL IDs. To assemble these IDs, a cross-search was performed between GO, String-DB, HUGO Gene Nomenclature Consortium [17], Mouse Genome Informatics [18], Rat Genome Database [19], and BioMart [20] databases. Genes that presented ID divergences among databases were manually curated or removed from our analysis. String-DB analyses were made with a 0.7 combined score and only interactions generated from experiments and databases were used. This is a medium to high stringency parameter.

### 2.2. Topology and Evolutionary Plasticity Analysis

Topologies of the receptors functional networks were analyzed by connectivity [*k*(*i*)] and clusterization [*c*(*i*)] indexes of their components. *k*(*i*) index is calculated by the number of neighbors that an *i* node has in a network, and *c*(*i*) by the equation

(1) $$\begin{array}{c} \;c\left(i\right)=\frac{2n_{i}}{k_{i}\left(k_{i}-\text{1}\right)}, \end{array}$$

which represent general interactivity of *i*'s neighbors, where *n*_*i*_ represents the number of their connections among each other. Evolutionary Plasticity Index (EPI) of the orthologous groups of these proteins was calculated by equation

(2) $$\begin{array}{c} \text{EPI}=1-\frac{H_{\alpha }}{\sqrt{D_{\alpha }}}, \end{array}$$

where *H*_*α*_ is the ortholog diversity in the eukaryotic tree, calculated using how many species the ortholog is found, and *D*_*α*_ is its abundance, calculated by the number of ortholog members found in each species [21]. Orthology data of these proteins was also gathered using String-DB. All statistical analyses were made using one-way ANOVA with Tukey's test. *k*(*i*) and *c*(*i*) indexes were compared by the Shannon diversity (*S*) of their distribution, using equation

(3) $$\begin{array}{c} S=-\sum p\ln p, \end{array}$$

where *p* is the probability of a value occurrence in any dataset. Entropy calculation was used in a complementary way in order to mathematically support or refute any observations in the connectivity and clusterization distribution behavior. In order to generate the graphical representations of the CR network, we have plotted String-DB interactions of all Gene Ontology groups proteins among each other using RedeR R package [22]. The list of all the genes analyzed in this work is presented in the Supplementary Material available online at http://dx.doi.org/10.1155/2014/696485.

### 2.3. Phylogenetic Analysis

Chemoreceptor genes sequences were gathered from the Chemosensory Receptor Database [23]. Alignments and trees were made with the MEGA 5.2 software [24], using, respectively, the Muscle alignment algorithm [25, 26] and the Tamura-Nei model [27]. Parameters used were the default for each algorithm. Branch reliability was calculated using bootstrap method. 100 bootstrap replications were performed for T1R, T2R, and VN and 50 replications for OR. For entropy analysis, we have calculated the Shannon diversity of the phylogenetic trees by subsetting each tree into *n* consecutives samples of *w* size, where *n* is the tree size and *w* is the maximum tree depth (i.e., number of levels). This was made to detect whether tree diversity was consistent throughout the entire tree radius. One-way ANOVA was used in order to compare these results. We have chosen *w* as the number of levels as a means for defining proportional windows for each tree.

## 3. Results

### 3.1. Differences between the Chemosensory Receptors and the Signal Transducing Machinery

**Figure 1:**
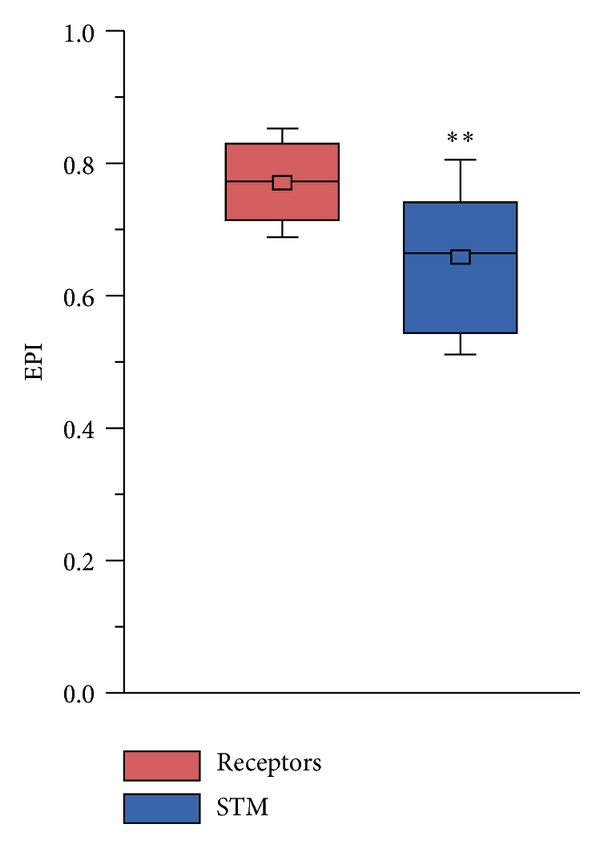
Mean EPI values of chemoreceptors (red) and signal transducing machinery (blue). The edges of the boxes indicate the upper and lower quartiles. The line at the center of each box indicates the median, the square represents the mean, and whiskers represent the standard deviation. Asterisks indicate statistically significant data (*P* < 0.001).

**Figure 2:**
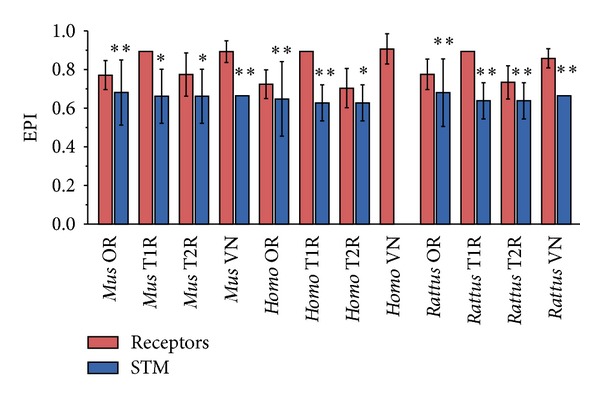
Mean EPI values of chemoreceptors families (red) and their respective signal transducing machineries (blue). Plasticity values are shown in the vertical axis and the different subgroups are listed on the horizontal axis. Whiskers represent the standard deviation. Statistically significant data are indicated by double (*P* < 0.001) and single (*P* < 0.05) asterisks.

We have calculated separately the Evolutionary Plasticity Index (EPI) [21] of the CR genes and their signal transducing machinery (STM). We have observed that CR genes as a whole have significantly higher plasticity values than the STM (Figure 1), indicating that CR genes have a broader ortholog distribution than the STM, meaning that the latter was subject to less variation during the course of evolution. To further corroborate these findings, we have compared each CR family separately to its signal transducing machinery. We have found that, in all cases but one, the EPI of each CR family was significantly higher than its STM (Figure 2). The exception was the human vomeronasal (VN) genes, which lack their STM due to the loss of the TRPC2 channel [28, 29].

**Figure 3:**
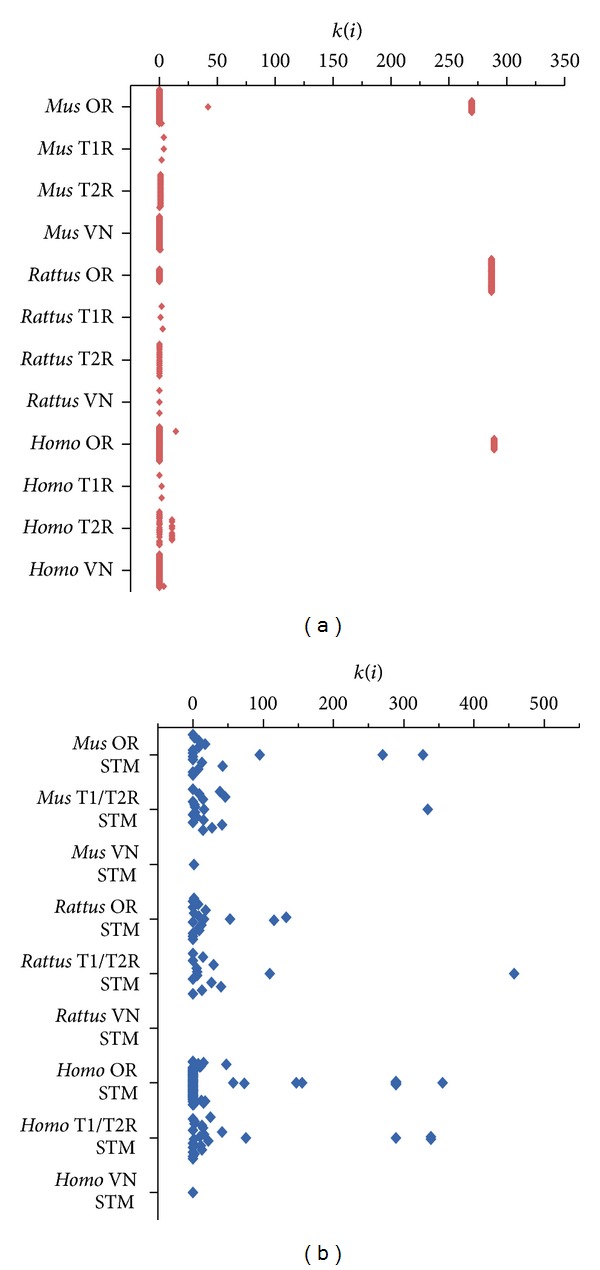
Connectivity values distribution for the chemoreceptors families (red) and their respective signal transducing machineries (blue). Values are shown in the vertical axis and the different subgroups are listed in the horizontal axis.

**Figure 4:**
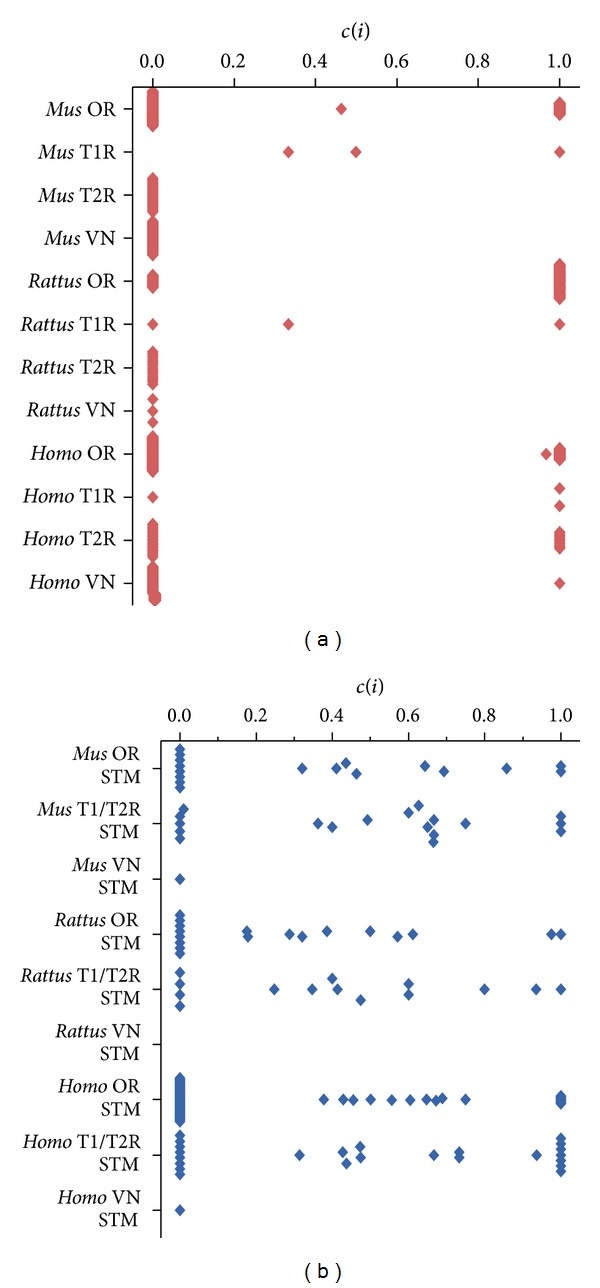
Clusterization values distribution for the chemoreceptors families (red) and their respective signal transducing machineries (blue). Values are shown in the vertical axis and the different subgroups are listed in the horizontal axis.

Next, we have compared the network topologies of each CR family and their STM. We have observed that most CR genes are functionally less connected than their STM. Most CR genes are connected only to their respective G proteins, indicating that they are located in the periphery of their functional networks (Figures 3 and 4). This assumption is further supported by analyzing the Shannon diversity of the connectivity and clusterization indexes. We have found that the STM has higher diversity values for these indexes (*P* < 0.05), suggesting that they occupy a broader range of niches in their network. Exceptions to this are some olfactory receptors, which presented higher connectivity and clusterization values among each other.

### 3.2. Differences among the Different Chemosensory Families

**Figure 5:**
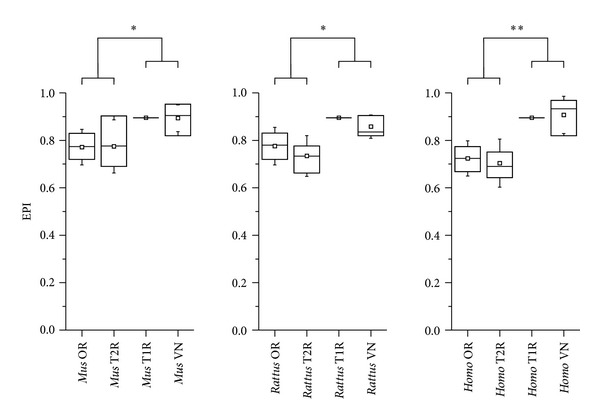
Mean EPI values of chemoreceptors families. Plasticity values are shown in the vertical axis and the different families are listed on the horizontal axis. The line at the center of each box indicates the median, the square represents the mean, and whiskers represent the standard deviation. Statistically significant data are indicated by double (*P* < 0.001) and single (*P* < 0.05) asterisks.

**Figure 6:**
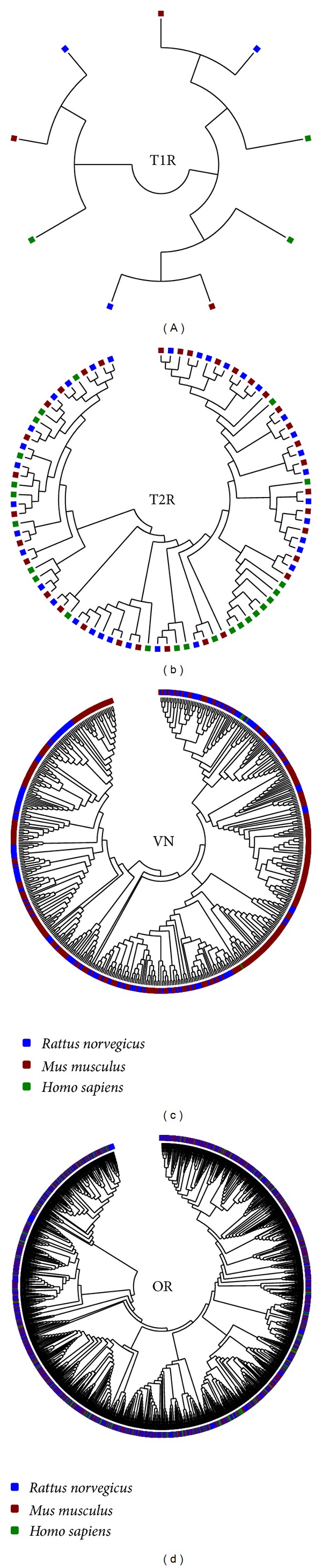
Reconstructed phylogenetic tree of the chemoreceptor families. Each square represents a CR gene. Blue, red, and green squares represent *Rattus norvegicus*, *Mus musculus*, and *Homo sapiens* genes, respectively. Phylogenetic trees were reconstructed with Tamura-Nei model. T1R: type I taste receptors; T2R: type II taste receptors; VN: vomeronasal receptors.

We have compared the EPI of the different CR families with themselves in order to identify differences in their orthologs distribution. Due to lack of data regarding the V1R and V2R, we have considered these genes as a single group in our analysis (VN). Strikingly, we have observed that CR families can be sorted in two groups regarding their plasticity. The OR and T2R have significantly lower plasticity than the T1R and VN in the three mammals we have studied, indicating that they had evolved under different constraints in these species (Figure 5). To further assess these differences, we have reconstructed the phylogenetic relationships among each CR family. We have observed that the OR, T1, and T2 genes form branches preferentially with their orthologs in other species, whereas the VN genes branches with their inparalogs (Figure 6). These results are further supported by calculating the Shannon diversity index stepwise for each CR tree. We have found that the VN tree had significantly lower diversity values (*P* < 10^−16^) than the other CR, suggesting that the VN genes are less conserved than the other CR. The original trees with bootstrap replications confidence values can be found in the Supplementary Material.

### 3.3. The Functional Organization of the CR Genes Network

**Figure 7:**
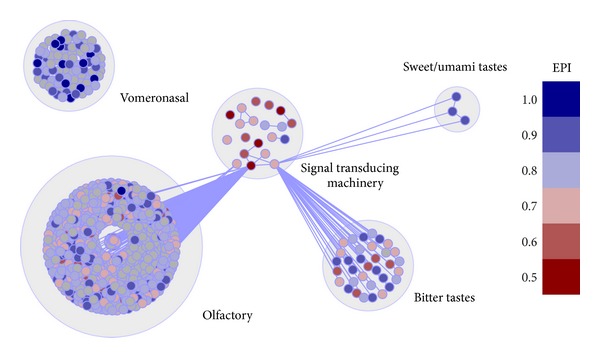
Graphical representation of *Mus musculus* chemosensory network. EPI values are plotted on each node by a color scale. Higher plasticity is indicated by bluish colors and lower plasticity by reddish colors. The other networks are not shown in this paper. Nodes represent protein coding genes and edges, functional interactions.

Finally, we have reconstructed the CR genes network in order to visualize its functional organization. We can observe that even though they form completely separate functional clusters, all the CR families, with the exception of VN, share the same STM cluster (Figure 7). This indicates that the STM machinery is essentially the same in every CR cell type.

## 4. Discussion

Chemosensory perception is one of the most important systems for appraisal of the environment. It is of vital necessity to every organism that the chemical species detected by each chemoreceptor are tuned to tastes or odorants which bring meaningful information from the outside world. Unlike physical sensory modalities, whose stimuli nature is constant (e.g., light, sound), chemical perception may be subject to radical changes in very short time windows. For instance, some plants are able to change their repertory of toxic secondary compounds in just a few generations [30], forcing herbivorous species that can potentially ingest these compounds to keep equally updated their ability for detecting these toxins. From an evolutionary point of view, this means that the genes coding these receptors must have a more relaxed behavior in order to accommodate novelties in the environment.

When comparing all CR genes to their STM, we have observed that CR have higher evolutionary plasticity values, suggesting that they were more subject to variation in the course of evolution than the STM. This indicates that the STM has remained relatively unchanged since its appearance, while the receptors themselves were free to experiment with the environment. By analyzing the network topology of the CR and STM, we have observed that CR occupy a peripheral position in their functional network. It has been proposed that proteins located in the periphery of their respective functional networks have elevated propensity to duplicate and undergo positive selection [31, 32]. This happens because poorly connected and loose clusters are able to more efficiently accommodate evolutionary novelties such as gene duplications, deletions, and changes of function, and thus they become the “evolutionary motors” of their biological networks [33–35]. D'Antonio and Ciccarelli have recently demonstrated evidences supporting this assertion [36]. In their paper, these authors have thoroughly analyzed network properties, sequences, and orthology data from *E. coli*, yeast, fly, and human. They observed that genes acquired during evolution encode less connected and less central proteins that are subject to more duplication events. Conversely, it has been observed in other types of signal transducing cascades that the receptors are more constrained than the intermediate elements of their networks [37–40]. These studies, however, were made with pathways such as those of insulin/TOR, which integrate information from inside the organism. As corporeal composition remained relatively the same throughout vertebrate evolution, intra- and extracellular components are not subject to radical variation, making necessary that internal signal transducing cascades must be more tightly constrained in order to consistently maintain their behavior. The environment, however, is constantly subject to changes, and the chemoreceptors cannot be too tightly constrained in order to accommodate these fluctuations. Our data support that CR are a special case of signal transducing pathways that have loosely constrained receptors.

Our subsequent insight into CR evolution was made when comparing the receptors with themselves. We have observed striking evidences suggesting that the vomeronasal receptors are less constrained than the other CR families. First, their plasticity is significantly higher than the other CR, suggesting that this gene family was probably more subject to duplications and deletions than the other receptors. This is further supported by their phylogenetic tree, which is grouped by inparalogs rather than orthologs, suggesting that these genes have arisen from recent duplications and, therefore, are probably less constrained. This finding is similar to what Grus and Zhang observed when studying the dynamics of vomeronasal and olfactory receptors in vertebrate species [41]. Lastly, we have observed that their functional network is completely detached from the other receptors, making them the most peripheral CR. From an evolutionary point of view, one would be tempted to think that the VN code the least important CR in terms of individual survival. The T2R are responsible for detection of bitter tastes. In general, these tastes are typically associated with toxic nitrogenated compounds, such as alkaloids and amines [42]. The perception of these toxins is a major issue in the survival of any organism that has chances of ingesting them. Equally important to their survival is the detection of food, predators, and members of the same species by OR. Conversely, the VN genes likely give clues about potential reproductive partners by detecting genetic likeness and even immune compatibility [43, 44]. These characteristics, albeit very important to long term adaptation and survival of the species as a whole, are not a major issue in direct survival of the individual.

An apparent contradiction in our analysis was the case of the T1R, which code sweet and *umami* receptors. From our phylogenetic analysis, these receptors are tightly constrained. All three species have the same number of these receptors, each branching more closely with its orthologs in other species rather than the others of the same species. This finding is supported by an earlier analysis that found the same pattern in all vertebrate species [45]. However, by their ortholog distribution, we have found high EPI values. These receptors are grouped in the KOG1056 group, which encompasses 1790 proteins in 52 species, with most varied functions (e.g., bride of sevenless, a homeotic gene). The high-plasticity values of T1R family are owed to the comprehensive reach of this orthologous group, suggesting that these receptors are constrained members of a larger and more dynamic family of proteins. Albeit instigating, these assumptions can only be confirmed with further in-depth study of this interesting orthologous group.

From a systems perspective, we have found evidences that the CR evolved through duplication events that resulted in gain of function. We have observed that all CR families share the same STM cluster, suggesting that the latter is an older transducing core that was reused in several cell types. The CR, on the other hand, are specific and only expressed in their appropriate cell type. The only CR family that diverges from this behavior is the vomeronasal receptors, which were adapted to convey their signal directly to an ion-channel. This deviation may be the reason why these receptors are under different evolutionary constraints.

Our results suggest that genes coding chemoreceptors were subject to more variation in the course of evolution than those coding signal transducing machinery, reflecting their distinct functional roles in organisms. We have also found significant variation even among the different receptor modalities, suggesting, for the first time to our notice, that olfactory and bitter taste receptors are, albeit less constrained than the transduction machinery, more conserved than vomeronasal receptors. These differences are due to the distinct ecological roles played by the receptors, with the low-plasticity olfactory and bitter taste receptors taking major part in direct survival of the organism, whereas high-plasticity vomeronasal receptors contribute to overall adaptation of the species. Sweet/*umami* receptors cannot be analyzed by their orthologous distribution alone due to the large variability of their ortholog group, and further studies are needed in order to understand the selective pressures imposed on them. We believe that the chemoreceptor networks case is illustrative to demonstrate the generation of novelties through evolutionary tinkering. During the course of evolution, the chemosensory cells generated novel receptor clusters probably by duplicating older ones in order to perceive different sensory inputs. Even among these clusters, there is a great deal of evolutionary experimentation, so that the organisms can be kept up to date with their environment. The signal transduction machinery and other information pathways, however, remained essentially the same throughout generations.

## Supplementary Material

This supplementary material contains:** 1) A summary of all genes used in our analyses, with their respective orthology and topology information 2) The tree files used to generate Figure 6
Please refer to the readme file further information.
